# Insights into germination, physiological, and molecular changes in aniseeds induced by magnetic fields

**DOI:** 10.1038/s41598-026-54832-2

**Published:** 2026-06-23

**Authors:** Ahmed ElFatih ElDoliefy, Alia Amer, Eslam S. ElShahed, Haitham S. Mohammed, Aml Shahin, Fatma M. Kamel, Mariam Sabry, Moreen Yousef, Mira Essam, Klara Soliman, Monica Sobhy, Batool Samir

**Affiliations:** 1 Laboratory of Applied Plant Breeding and Microbial Biotechnology (APBMB), Department of Plant Molecular Biology, Agricultural Genetic Engineering Research Institute (AGERI), Agricultural Research Center, Giza, Egypt; 2 Medicinal and Aromatic Plants Research Department, Horticulture Research Institute, Agricultural Research Center, Giza, Egypt; 3 Biophysics Department, Faculty of Science, Cairo University, Giza, Egypt; 4 Biotechnology/Biomolecular Chemistry Program, Faculty of Science, Cairo University, Giza, Egypt

**Keywords:** Magnetic field, Seed priming, *Pimpinella anisum* L., Seed germination, Antioxidant enzymes, Gene expression, Low-frequency, Physiology, Plant sciences

## Abstract

Improving seed germination is essential for enhancing crop establishment under increasingly variable environmental conditions associated with climate change. Magnetic field (MF) treatment represents a clean, non-chemical, and sustainable seed-priming approach; however, frequency-dependent biological responses remain insufficiently understood. This study investigates the responses of aniseed (*Pimpinella anisum* L.) to static (DC) and low-frequency alternating magnetic fields (5, 10, and 15 Hz) across different exposure durations. Germination parameters (percentage, speed, vigor index), physiological traits, and activities of key hydrolytic and antioxidant enzymes (α-amylase, protease, catalase) were assessed. Furthermore, the molecular expression of the stress-responsive superoxide dismutase (SOD) and the cytoskeletal actin genes was analysed. MF significantly enhanced germination percentage (up to a 25% increase), mean germination time, and vigor indices compared to the untreated controls. Physiologically, treated seedlings exhibited higher antioxidant defense levels. At moderate frequencies, catalase activity increased, while α-amylase and protease were markedly elevated at higher frequencies, enabling reserve mobilization and stress tolerance. At the molecular level, *sod* transcripts were down-regulated across all MF treatments compared to the control, indicating a functioning oxidative stress response. These findings demonstrate that MF frequency modulates the integration of physiological (enzyme-driven metabolism) and molecular (antioxidant gene regulation) pathways to optimize during aniseed germination. This research provides mechanistic insights and presents low-frequency MF as a viable seed priming for sustainable crop improvement under dynamic environments.

**Supplementary Information:**

The online version contains supplementary material available at 10.1038/s41598-026-54832-2.

## Introduction

In agriculture, seeds of superior quality promote uniform and rapid seedling emergence, more plant establishment, higher densities, and ultimately higher production^1–3^. One of the distinguishing features of high-quality seeds that is essential for successful seedling establishment and subsequent achievement in the later phases of plant development is seed germination^4^. The common anise (*Pimpinella anisum* L., Apiaceae) plant is also recognized as aniseed and sweet cumin^5^. It flourishes in the Middle East, West Asia, and the Eastern Mediterranean region^6^. Anise is an essential spice characterized by fruit-like seeds^7^. The fruits and their essential oils are noteworthy for their economic use in food, perfumery, pharmaceutical, and traditional medicine.^8,9^. Conversely, anise plants have small, irregular seeds with low vitality, vigor, and nutrient stores as well as poor quality, which in turn influences the plant density and emergence, ultimately affecting plant production^10^. Additionally, high-quality seeds produce seedlings with more vigor and resilience to adverse environmental conditions^11^.

Seed priming can enhance the processes of germination and seedling growth^12^. Although chemicals are widely used for treating seeds, they contain environmentally harmful compounds^10,13^. Therefore, research efforts are focused on developing technologies that can enhance crop production and seedling vigor by exposing seeds to physical agents. Scientists are striving to make this approach more efficient and friendly to the environment^14^. For this aim, the magneto priming seed was proven to be efficient, economical, secure, and enhanced plant growth and crop yield^15^. Although the biological stimulators can boost plant growth, crop yield, and resistance, magnetic fields (MF) have more benefits, lacking the harmful residues of the chemical stimulators^16^. The benefits of applying magnetic field stimulations to seeds have been documented in the literature on various crops such as palm oil (*Elaeis guineensis* Jacq)^17^, bean (*Phaseolus vulgaris* L.)^18^, onion (*Allium cepa* L.) and lettuce (*Lactuca sativa* L.)^19^, brown rice (*Oryza sativa* L.)^20^, and medicinal sage (*Salvia officinalis* L.)^21^.

To explain MF effects, scientists have developed a few theories^22,23^. Some of these theories proved that MF can enhance the seed’s vigor, and cause some biochemical processes, including the enzymes such as protease and α-amylase, proteins, and antioxidant enzymes like SOD^24–27^. Furthermore, the production of pharmaceutically high-value secondary metabolites was generally enhanced by MF and supported by the combined application of electro-MF and nanoparticles (SiO_2_) that significantly enhanced protein content and the activity of antioxidative enzymes, including SOD, CAT, and peroxidase^28^. However, there are currently just theories about MF action and no evidence-based study of how MF affects plants. While many previous studies have focused on the static magnetic field (DC) and its influence, few studies systematically explore the possible effects of the different alternating current frequencies in combination with various exposure times.

Consequently, the main goal of this research is to thoroughly assess aniseed behavior when exposed to different MF frequencies, including DC (0 Hz), 5 Hz, 10 Hz, and 15 Hz, over varying durations. This is accomplished by analyzing changes in germination, seedling vigor, and the underlying biochemical and molecular mechanisms. Based on these objectives, the current study tested two hypotheses: first, that aniseed germination and antioxidant responses are differentially regulated by a low-frequency magnetic fields in a frequency-dependent manner; and second, that antioxidant enzyme activities and gene expression profiles demonstrate the optimal MF frequency across different exposure times. Furthermore, this study aims to demonstrate how these treatments enhance seed vigor by accelerating the metabolic reserve without inducing excessive oxidative stress. This comprehensive evaluation research offers a deeper understanding of how geophysical forces, particularly magnetic fields—which are a clean and sustainable approach—interact with plant seeds. Ultimately, this research seeks to establish a precise and reproducible protocol for applying MF for specific durations to improve germination, as a vital step toward boosting productivity and advancing global food security.

## Results

The assessment of enzyme activity and *sod* gene expression was specifically limited to seedlings resulting from the 90-min magnetic field exposure. This choice was justified by the fact that this duration produced the maximum G% and superior shoot/root development by the 3rd week across all magnetic frequencies.

### Effects of static and low-frequency magnetic fields on aniseeds germination performance

#### Effects of static magnetic field (DC) on germination traits of aniseeds

**Fig. 1:**
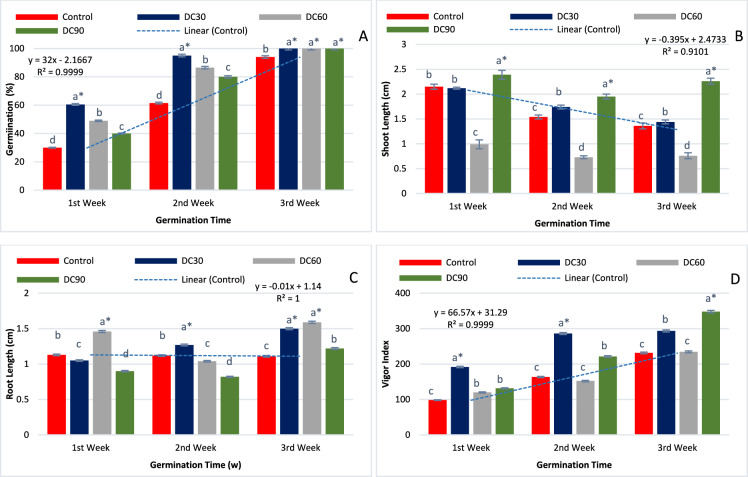
Effects of static magnetic field (DC) on germination traits of aniseeds. (**A**) Germination, (**B**) shoot length, (**C**) root length, (**D**) Vigor index. Data expressed as mean (± S.D), different letters are significantly different (*p* < 0.05) based on Duncan’s multiple range test.

The DC MF was effective on G% and other germination parameters, with consistent performance over 3 weeks (Fig. 1). However, all DC treatments significantly accelerated the percentage of germination during the first 2 weeks, ultimately achieving a maximum of about 100% by the 3rd week (Fig. 1A). Interestingly, the growth parameters presented a nuanced and treatment-specific response to the DC MF application. By the 3rd week, DC at 90 min of exposure consistently produced the highest shoot length (Fig. 1B) and vigor index (Fig. 1D). Notably, shoot length under DC 30 recorded a gradual decline from the first to the 3rd week. However, this treatment appeared to inhibit root extension during the initial germination stages compared to the control and other treatments (Fig. 1C). In contrast, the application of DC for 60 min exhibited a significant correlation with root development, peaking in the 3rd week. In general, the data in Fig. 1 showed that while lower or moderate doses (DC 30 and DC 60) optimally support root development, DC 90 application produces the most robust long-term enhancement to total seedling vigor and shoot growth over time.

#### Effects of 5 Hz low-frequency magnetic field on germination traits of aniseeds

**Fig. 2:**
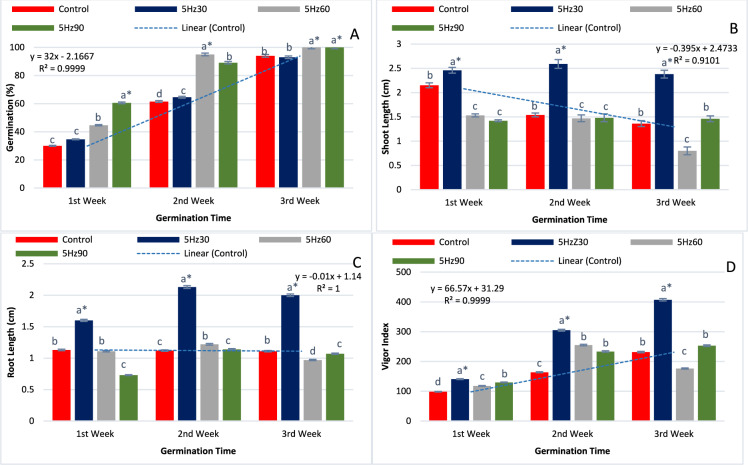
Effects of 5 Hz low-frequency magnetic field on germination traits of aniseeds. (**A**) Germination, (**B**) shoot length, (**C**) root length, (**D**): Vigor index. Data expressed as mean (± S.D), different letters are significantly different (*p* < 0.05) based on Duncan’s multiple range test.

Based on data illustrated in Figs. 2 and 5 Hz at 30 min of exposure consistently outperformed both the control and other treatments across most growth parameters. While all 5 Hz treatments achieved near total germination by the 3rd week, the 5 Hz 60 and 5 Hz 90 treatments demonstrated a significantly higher germination velocity during the first 2 weeks compared to the control (Fig. 2A). However, this early surge didn’t translate into long-term development for the higher doses. Instead, 5 Hz 30 emerged as the optimal treatment, providing the maximum shoot and root length throughout the entire 21-day period. In contrast, the 60 and 90 min exposure occasionally exhibited inhibitory effects, with growth rates falling below the control levels (Fig. 2B, C). These trends culminate in the vigor index (Fig. 2D), in which the 5 Hz 30 treatment exhibited the highest peak, reaching approximately 400 by the 3rd week; however, the other treatments, including the control, lagged considerably behind.

#### Effects of 10 Hz low-frequency magnetic field on germination traits of aniseeds

**Fig. 3:**
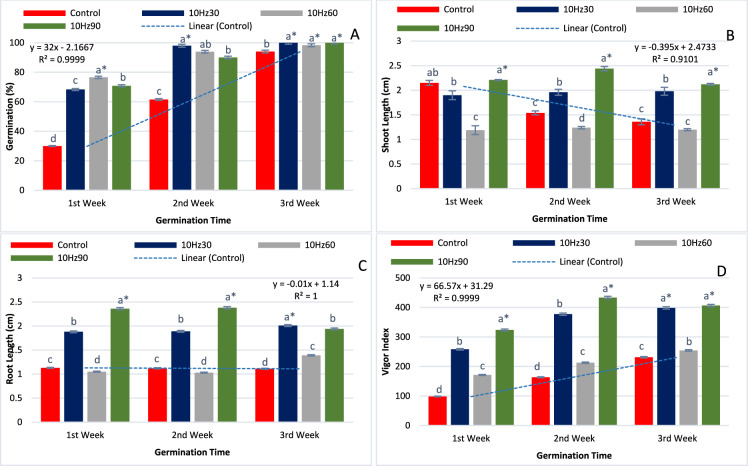
Effects of 10 Hz low-frequency magnetic field on germination traits of aniseeds. (**A**) Germination, (**B**) shoot length, (**C**) root length, (**D**) Vigor index. Data expressed as mean (± S.D), different letters are significantly different (*p* < 0.05) based on Duncan’s multiple range test.

The data in Fig. 3A show that all 10 Hz treatments significantly increased germination percentage compared with the control, with 10 Hz 30 and 10 Hz 90 treatments reaching their maximum values by the 2nd week. Whilst the length of shoot exhibited more variability, markedly with control, presenting a downward trend over time, the 10 Hz 90 consistently produced the maximum length of shoot and root across the 3 weeks (Fig. 3B, C). This superior performance is synthesized in the vigor index (Fig. 3D), where the 10 Hz 90 significantly outperforms the other treatments, reaching a stable peak near 450 by the 3rd week.

#### Effects of 15 Hz low-frequency magnetic field on germination traits of aniseeds

**Fig. 4:**
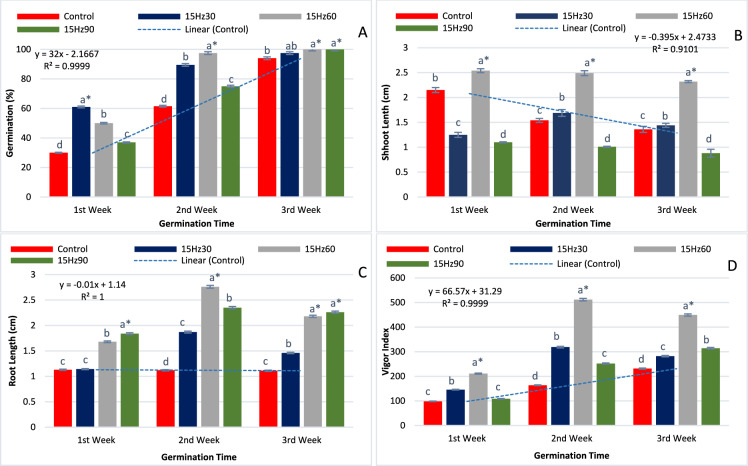
Effects of 15 Hz low-frequency magnetic field on germination traits of aniseeds. (**A**) Germination, (**B**) shoot length, (**C**) root length, (**D**) Vigor index. Data expressed as mean (± S.D), different letters are significantly different (*p* < 0.05) based on Duncan’s multiple range test.

Generally, 15 Hz 60 treatment regularly outperformed other treatments, including control, across most growth parameters, with a pronounced peak in the 2nd week (Fig. 4). Specifically, Fig. 4A illustrates that while all treatments eventually achieved 100% germination by the 3rd week, the treated aniseeds with 15 Hz 30 showed significantly faster initial germination than the control. However, as seen in Fig. 4B, C, the shoot and root length highlight a distinct advantage for the 15 Hz at 60 min exposure, which produced the most robust growth throughout the study. These trends are reflected in the vigor index (Fig. 4D), where the 60 min achieved its highest value in the second week. Although the 15 Hz for 90 min frequently outperformed noticeably worse shoot length, indicating that prolonged exposure may eventually impede some elements of plant development.

#### Interrelationships among germination parameters under magnetic field treatments

All parameters assessed for the germination of aniseeds showed significant variation in at least two sources of variation. The experiment was applied to assess the variation due to the different effectors, including the magnetic field application (Mag), the duration time (Dur), and the number of days required to reach completely germinated plantlets (Table 1). For the germination percentage (G%), the experiment showed significant variation due to the magnetic treatment (Mag) and the interaction between magnetism and the duration time (Mag*Dur) of magnetic application (*p* ≤ 0.001). For the shoot length (SL) parameter, the experiment showed significance due to the duration time (Dur) of magnetic treatment, the days required for germination, and the interaction (Mag*Dur). For the root length (RL) parameter, the experiment showed significance due to the magnetism (Mag) and the interaction (Mag*Dur) with duration time. For the VI parameter, the experiment showed significance due to magnetism (Mag), duration (Dur), and the interaction between them (Mag*Dur). All assessed parameters of germination showed a positive correlation with the various strength values (Table 2). The G% was low, associated with both SL and RL of 0.08 and 0.06, respectively, at a significance level of *p* ≤ 0.05. The G% was moderately associated with the VI (0.48^**^) at high significance (*p* ≤ 0.01). The SL parameter was moderately low, associated with RL (0.31 at *p* ≤ 0.05), while highly significant (*p* ≤ 0.01) with VI (0.74^**^). The RL parameter was highly associated (0.72^**^) and significant (*p* ≤ 0.01) with VI as well. This implies that the high significance is mainly due to the VI parameter (Table 2).

**Table 1:** Analysis of variance among the various magnetic treatments and durations applied to aniseeds.

SOV	G%				SL				RL				VI			
	*d.f*	*M.S*	*V.R*	*F pr*	*d.f*	*M.S*	*V.R*	*F pr*	*d.f*	*M.S*	*V.R*	*F pr*	*d.f*	*M.S*	*V.R*	*F pr*
Mag	4	1581.1	11.13	< 0.001	4	0.16	0.58	0.68	4	2.93	10.61	< 0.001	4	62,096	8.30	< 0.001
Dur	3	92.9	0.65	0.58	3	1.52	5.74	0	3	0.24	0.87	0.46	3	24,199	3.23	0.03
Days	2	96.5	0.68	0.51	2	0.78	2.94	0.059	2	0.33	1.18	0.31	2	1397	0.19	0.83
Mag*Dur	5	532.7	3.75	0	5	5.37	20.22	< 0.001	5	2.6	9.42	< 0.001	5	60,584	8.10	< 0.001
Mag*Day	8	214.6	1.51	0.17	8	0.16	0.62	0.76	8	0.18	0.65	0.73	8	5127	0.69	0.7
Dur*Day	6	37.1	0.26	0.95	6	0.04	0.15	0.99	6	0.01	0.04	1	6	464	0.06	1
Mag*Dur*Day	10	150.4	1.06	0.4	10	0.09	0.35	0.97	10	0.16	0.58	0.82	10	4037	0.54	0.86
Residual	76	142.1			76	0.27			76	0.28			76	7481		
Total	116				116				116				116			

G%, germination percentage. SL, shoot length. RL, root length. VI, vigor index. SOV, sources of variation. *d.f.*, degrees of freedom. M.S., means square. V.R. variation. *Fpr*. probability of Fisher-value. Mag, magnetic treatments. Dur, duration of magnetic treatment. Days, the interval times the data collected in. *Represent the interactions among the various sources of variation.

**Table 2:** Pearson correlation among the various assessed germination parameters.

VI	RL	SL	G			
0.48**	0.056	0.08	1	Pearson Correlation	G	
0.00	0.735	0.62		Sig. (two-tailed)		
39	39	39	39	N		
0.74**	0.305	1	0.083	Pearson Correlation	SL	
0.000	0.059		0.617	Sig. (two-tailed)		
39	39	39	39	N		
0.72**	1	0.31	0.056	Pearson Correlation	RL	
0.000		0.06	0.735	Sig. (two-tailed)		
39	39	39	39	N		
1	0.72**	0.74**	0.479**	Pearson Correlation	VI	
	0.000	0.000	0.002	Sig. (two-tailed)		
39	39	39	39	N		

**The correlation is significant at the 0.01 level (two-tailed).

For the G%, the significant source of variation was due to the MF application and the interaction between the MF and the duration time. By dissecting these differences using the LSD value (11.19), we found that the 0 Hz was effective with the DC, 5, 10, and 15 Hz, while the 10 Hz was effective with all MF strengths. At the level of interaction between the MF and the duration time, we found that at the duration of 30 min, the significant differences were between the 0 and 5 Hz on one side and the DC, 10, 15 Hz on the other side (Supplementary file). At the duration of 60 min, the 0 Hz showed a significant difference [15.9, 19, 28.8, and 20.3% (LSD = 11.19)] with the DC, 5, 10, and 15 Hz; respectively. Likewise, the DC showed a significant difference (12.9%) with the 10 Hz. At the 90-min duration, the 0 Hz showed a significant difference [15.6, 21.1, 26.7, and 11.3%, (LSD = 11.19)] with the DC, 5, 10, and 15 Hz; respectively. Likewise, the 15 Hz showed a significant difference (15.4%) with the 10 Hz.

For the RL parameter, two significant sources of variation were observed (due to the MF and MF*Duration). By dissecting these differences using the LSD value (0.493), we found that the MF application using the 0, DC, and 5 Hz showed significant differences with the 10 and 15 Hz. However, at the source of variation due to the interaction between MF and duration, we found that at the 30-min duration, the 0 Hz showed significant differences with the 5 and 10 Hz (0.79 and 0.81 cm, respectively), and the DC showed significant differences with 5 and 10 Hz (0.64 and 0.65 cm, respectively). At the 60-min duration, only the 15 Hz showed significant differences with 0, DC, 5, and 10 Hz (1.08, 0.84, 1.10, and 1.05, respectively). At the 90-min duration, the 10 Hz showed significant differences with the 0, DC, and 5 Hz (1.107, 1.248, and 1.246; respectively), and the 15 Hz showed significant differences with the same MF strength (1.03, 1.172, and 1.17; respectively). Other parameters and the observed significant differences between MF, duration, time of the experiment (days), and the interaction among them were detailed in the Supplementary file.

#### Magnetic field-induced modulation of germination-related enzyme activities in anise seedlings

**Fig. 5:**
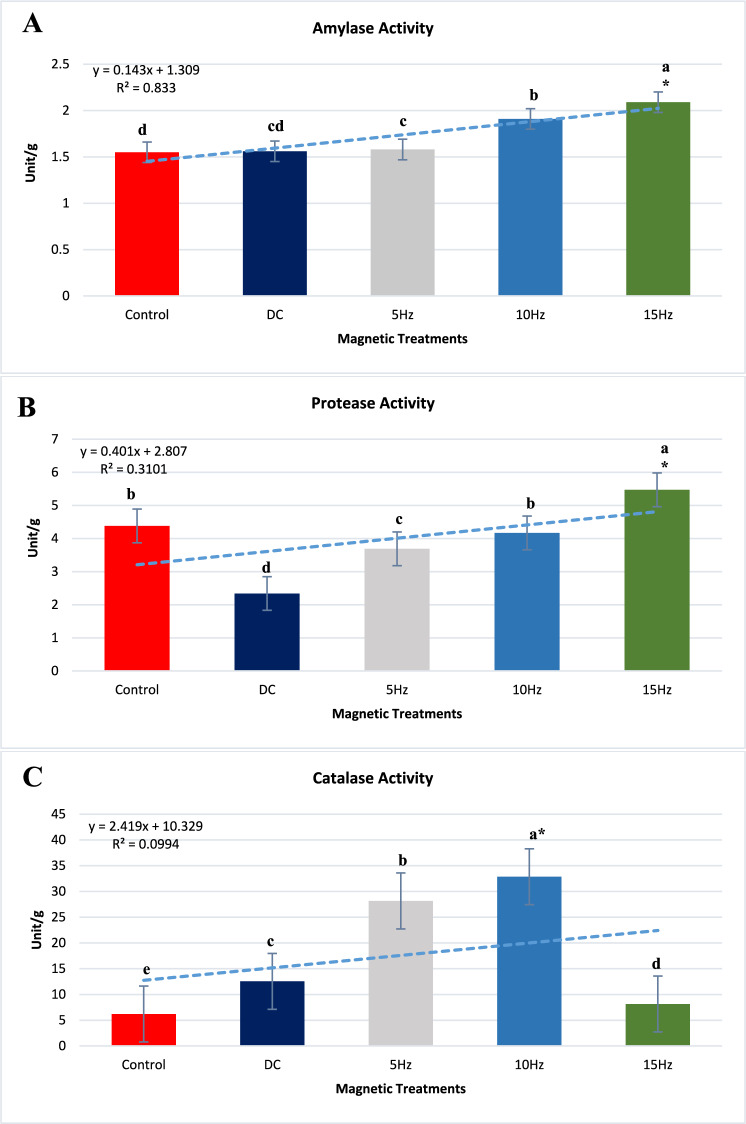
Physiological enzymes in germinated aniseeds under the four MF treatments applied for 90 min. (**A**) amylase activity. (**B**) Protease activity. (**C**) Catalase activity. Enzyme activities are expressed as U/g of fresh weight. Data expressed as mean (± S.D), different letters are significantly different (*p* < 0.05) based on Duncan’s multiple range test.

As presented in Fig. 5, the application of magnetic fields for 90-min of duration to the aniseeds caused a significant change in the physiological enzyme activity of the germinated seedling. The amylase, protease, and catalase enzyme activities were markedly higher in the aniseeds treated with the different magnetic fields compared to the untreated seeds. The amylase and protease activity was notably higher in the emerging seedlings of magnetized seeds by 15 Hz for 90 min (Fig. 5A, B). At the same time (90 min), catalase activity presented the most significant increase in emerging seedlings from magnetized seeds at 10 Hz (Fig. 5C).

#### Influence of magnetic treatments on the gene expression of superoxide dismutase and actin

**Fig. 6:**
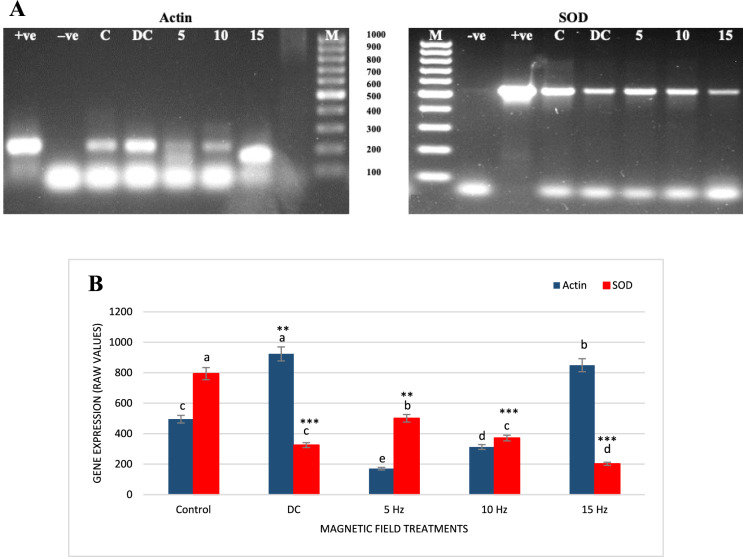
Influence of MF on the expression of antioxidant enzyme and housekeeping genes. (**A**) Amplified reverse transcripts of actin and superoxide dismutase (*sod*) genes expressed under the four MF treatments applied for 90 min on the aniseeds. −ve, H_2_O sample. + ve, the amplified fragment using the DNA. The used DNA marker (M) is the Thermo Scientific 100 bp ruler. (**B**) Gene expression of raw values of *sod* and actin genes of aniseeds after 90 min under the MF treatments (DC, 5, 10, and 15 Hz intensities). Data expressed as mean (± SD) letters noted significant differences (*p* < 0.05) based on Duncan’s multiple range test. * indicates significance at *p* ≤ 0.05; **indicates significance at *p* ≤ 0.01; ***indicates significance at *p* ≤ 0.001.

The gene expression was presented in Fig. 6A, where superoxide dismutase (*sod*) was represented by the fragment sizes of 580 bp in all MF treatments for 90 min, as well as in the control. The actin was presented at fragment sizes ranging from 165 to 198.9 bp (Fig. 6A). No fragments were produced from the negative (−ve) reactions of the water sample. Both genes (*sod* and actin) exhibited differential expression across the various MF treatments applied to the aniseeds. The gene expression of *sod* was significant (*p* = 0.002–0.015) under all MF treatments, while that of actin was only significant (*p* = 0.032) under the DC. Compared to the control, the greatest change in *sod* expression was observed at 5 Hz (0.6-fold; *t* = − 4.32; *p* = 0.015) of MF treatment. The second highest level of *sod* expression was observed at the 10 Hz (0.5-fold; *t* = − 6.19; *p* = 0.006) of MF treatment. The gene expression of *sod* was observed as down-regulation under all MF treatments (Fig. 6B). This suggests that ROS levels were lowered in response to the MF treatment. This resulted in an overall decrease in the slope of *sod* expression with increasing MF intensity relative to the control. The actin showed a fluctuating expression pattern of up- and down-regulation. This suggests conformational adaptation to the cell structure and skeleton, along with the change in MF. Compared to the control, the greatest change in actin expression was observed at the DC and the 15 Hz (1.9- and 1.7-fold; *t* = 3.25 and 2.32; *p* = 0.032 and 0.78; respectively) of MF treatment. Despite the insignificance (*p* = 0.078–0.32), the overall slope of the actin expression increased as the MF treatments increased (5–15 Hz, Fig. 6B).

## Discussion

Enhancing seed germination and raising the metabolic rate are key functions of MF bio-stimulation. The current study demonstrates how MF intensities and exposure time affect aniseed germination, physiological, and gene expression. Exposing aniseeds to various MF intensities resulted in changes in germination dynamics and seedlings’ vigor (Figs. 1, 2, 3, 4). Similar results have been reported for sunflower (*Helianthus annuus* L.)^29^, golden pea (*Lathyrus chrysanthus Boiss*)^30^, pepper (*Capsicum annum* L.) and okra (*Abelmoschus esculentus* L.)^31–33^, sorghum (*Sorghum bicolor* L. Moench)^34^, faba bean (*Vicia faba* L.)^35^, soybean (*Glycine max* L. Merr)^36^, maize (*Zea mays* L.)^37^, wheat (*Triticum aestivum* L.)^38^, cumin (*Cuminum cyminum* L.)^39^, and chinese cabbage (*Brassica rapa* L.)^40^. A linear link between seed germination and MF intensity was discovered by^41–43^. Our results demonstrate a strong synergistic correlation between germination kinetics and seedling development across all MF treatments (Figs. 1, 2, 3, 4). While the germination percentage achieves a plateau of about 100% by the 3rd week, the vigor index represents a more sensitive indicator of treatment efficiency, highlighting the critical role of VI as a multidimensional metric of seed quality. This reflects the enzymatic efficiency and accumulated protein stores enabling the seedling to sustain superior growth rates as discussed by^44^. This confirms that seed vigor is not an extraordinary attribute, but a complex composite of rapid germination and biomass potential^45,46^. The rapid germination that occurred in the first 2 weeks is not just a temporal shift but is functionally correlated with the higher shoot and root length^47^. Consequently, this positive correlation suggests that MF establishes physiological progress, such as activation of enzymes, positioning the vigor index as a more comprehensive indicator of seed robustness^27,48^.

Notably, the growth parameters exhibited a duration-dependent response, with 90-min exposure under most MF frequencies consistently yielding the highest shoot length by the 3rd week. In contrast, the 30-min duration showed an initial stimulatory effect that gradually declined as germination progressed (Figs. 1, 2, 3, 4). This divergence could be explained by the hormetic threshold phenomenon, where shorter exposure provides only a transient metabolic “spark” that fails to sustain long-term vigor^49,50^. As discussed by Saletnik et al.^51^ and Afzal et al.^52^, the 90-min duration likely crosses a critical physiological threshold required for the stabilization of protective proteins and antioxidant signaling. Without reaching this threshold, the 30 min of MF exposure, seedlings may experience a metabolic ‘exhaustion’ phase or insufficient antioxidant cushioning to maintain accelerated growth rates through the later stages of development^53^. This highlights that MF bio-stimulation follows a dose–response relationship where exposure time is as critical as intensity for establishing permanent physiological progress^54^.

In the current study, specific hypotheses were developed to explain the impact of MF on seed germination and seedling growth, although the exact mechanisms remain uncertain. According to previous reports, MF exposure to seeds leads to biochemical changes and altered enzymes, impacting plant functions like photosynthesis, growth, mineral feeding, water uptake, and transport^24,55–60^. Ca^2+^ ion transport is a critical component of all plant functions, where changes in Ca^2+^ ion concentration and ionic densities can affect these activities. Similar observations have been proven by^27,61–63^. In addition, MF influences alterations in the physical characteristics of different solutes within plant cells, including cytoplasm^27,64^. Moreover, numerous studies have demonstrated that MF affects cellular metabolism and reproduction^65–68^. According to Souza et al.^69^ and Selim et al.^70^, the MF stimulates IAA, cytokinins, and GA synthesis, which encourages plant growth and cell division. In line with Dhawi, Shan, and Hafeez studies, increased hydrogen bonds in the molecules, as well as cell permeability and active energy in cell electrolyte solutions in MF-influenced seeds, can impact enzymatic activity, biochemical reactions, and physiological processes, thereby accelerating seed germination^27,71,72^.

In current research, catalase, amylase, and protease enzymes have been increased in seeds treated with different magnetic field treatments (Fig. 5). The changes in enzyme activity in seeds treated with magnetic fields were reported in different reviews^73–78^. Under MF stress, higher plants develop a complex network of antioxidant enzymes, utilizing both enzymatic and non-enzymatic antioxidants to survive and combat elevated ROS levels^79–84^. Plant SOD, metalloenzymes co-factored by metal ions (_Cu/Zn_SODs, _Fe_SODs, and _Mn_SODs), catalyze the dismutation reaction of superoxide anion [(O_2_^−^) to O_2_ and H_2_O_2_ to oxygen and water]. The _Cu/Zn_SOD can be found in the cytosol, chloroplasts, mitochondria, peroxisomes, and extracellular space, while the _Fe_SOD is found in the chloroplasts, and the _Mn_SOD is found in the mitochondria^85–91^. Therefore, _Cu/Zn_SOD is dominant in plant cells as it is the first and fastest-ready machinery to respond against ROS elevation.

The enhancement of α-amylase and protease activities in MF-treated seeds indicates accelerated mobilization of carbohydrate and protein reserves, such processes that are critical for energy supply and amino acids during early seedling growth^74^. Such metabolic activation is consistent with the observed improvements in germination performance and seed vigor. Increased metabolic flux during germination is commonly associated with elevated mitochondrial activity, which may lead to enhanced production of ROS^26^. ROS such as O_2_^−^ and H_2_O_2_ act as signaling molecules during dormancy release and germination, and antioxidant systems, including SOD, are transcriptionally and enzymatically adjusted to maintain redox homeostasis during stimulated germination^92,93^. In this context, the observed modulation of *sod* gene expression likely reflects a transcriptional adjustment aimed at maintaining redox homeostasis during MF-stimulated germination.

Responsive SOD activities have been reported in many plants exposed to stressful environments^83,94–97^. *sod* expression may infer spatial and temporal patterns in different plant tissues and developmental stages based on the differential cis-acting elements, the transcription factors, and the miRNA regulating those^98^. Additionally, SOD plays a crucial role in electron transport, photosynthesis, and signal transmission, as evidenced by the loss-of-function mutation in _Cu/Zn_*sod* of *Arabidopsis,* which results in decreased chloroplast size, chlorophyll content, and photosynthetic activity compared to the wild-type^99^. This influence of SOD on electron transport may be the reason behind its measured expression fold-change in anise plants treated with MF in the current study (Fig. 6B). Parallel, as the seeds of soybean were pretreated by MF, some enzymes (protease, amylase, catalase, SOD, and peroxides) were assessed, and the effect of MF for 2.2 to 19.8 s was evaluated. The results showed an increase in shoot formation, chlorophyll contents, and peroxidase activity^100^. These observations, along with our results on aniseeds, affirm the significant effect of MF on the activity of plant antioxidant enzymes in general and specifically the SOD.

Such records support the stressful effect of MF on plant cells, as evidenced by the induction of antioxidant enzymes to scavenge the excessive ROS produced because of the MF treatments. Supportively, our findings revealed that a potential fragment (580 bp—Fig. 6B) of a *sod1* isoform was expressed at varied intensities. More specifically, the MF effect (2.9–4.6 mT) on *sod* activity was investigated in soybean seed germination for 24- and 72-h treatments, where a significant and intensified increase (21.2%) was observed compared to the control samples^101^. Continually, the effect of a weak permanent MF (WPMF) of 185–650 µT was assessed on radish seedlings, where the low flux inhibited *sod* activity, while the higher flux activated it^102^. This observation may indicate a mechanism of interaction involving radical pairs, which are associated with the activation or inhibition of membrane lipid peroxidation and serve as potential targets for the PMF.

Controversially, the weak static MF (0–188 µT) suggested that cryptochromes may not be the only sensors of MF in plants and cryptochrome- and phytochrome-mediated plant responses can be modulated by the strength and the orientation of the local geomagnetic field^103^. This study has drawn some attention to the strength of MF as a modulator of the plant response. On the other hand, the exposure time and the static MF (SMF) intensities (0.44, 0.77, and 1 T) showed no significant differences in lettuce seedlings, though confirmed changes in SOD, CAT, and PX activities^104^. Additionally, two SMF intensities (10 and 28 mT) have improved the permeability of cellular membranes and activated the antioxidant system (SOD, CAT, and esterase) in coffee seeds, which may have an impact on speeding germination time and uniformity^105^. Generally, exposing lemon balm explants containing axillary meristem to MF (50 mT) showed the best (among 100 and 150 mT) enhancement of rosmarinic acid quantity, total phenol-flavonoid content, non-enzymatic antioxidant activity (IC_50_), stress-related enzymatic antioxidant activity (SOD and CAT)^106^.

It is proven that ROS disrupts normal metabolism, causing oxidative damage to membrane lipids, proteins, pigments, and nucleic acids^83,107,108^. Therefore, sequential detoxification of ROS is coordinated by antioxidant enzymes such as SOD^109^. Magnetized plasma in tomato roots showed a change in the catalytic activity of the dehydrogenase enzyme according to the increase in the current creating the MF^110^. The SOD isoenzymes in maize roots upon magnetized treatments suggested some dependence on seedlings’ age, where the most active enzyme was the _Cu-Zn_SOD (cytoplasm) over the _Mn_SOD (mitochondria)^111^. These observed differences in SOD isoenzyme activities in the current study (Fig. 5), as well as previous reports, can be related to the subcellular localization of these SOD isoforms in the cell upon the MF treatment^111,112^. Knowing that _Zn_SOD is sensitive to the increased quantities of H_2_O_2_ supports that the *sod1* gene may be the main to quenching ROS during oxidative stresses caused by physical forces in plant cells^111^. All these comments coincide with our current foundations of *sod1* isoenzyme expression according to the MF strength applied to the plant tissues. Interestingly, this isoform-preferential MF strength (association) observed on *sod* expressions/induction (Figs. 5, 6) could be influenced by the cellular compartment (cytoplasmic or mitochondrial) impacted by the MF forces.

With respect to all provided physiological and molecular data, we may assume that the magnetic field induces a dose- and frequency-dependent oxidative and metabolic responses (reprogramming) in germinating anise (*Pimpinella anisum* L.) seeds. At the cellular level, the increase in catalase activity at 5–10 Hz (28.16–32.86 vs. 6.21 in control), along with the altered *sod* expression, reflects an enhanced ROS and compensatory antioxidant responses triggered by the magnetic stimuli^64,113,114^. This means that the MF acted as an abiotic stressor targeting the ROS and providing a non-linear and frequency-specific response. The down-regulation of *sod* expression under the DC and the higher frequencies coupled with strong catalase induction implies a shift from superoxide detoxification toward hydrogen peroxide-centered signaling. This pattern of antagonistic regulation of *sod* and catalase suggests redox fine-tuning, which is consistent with a controlled ROS signaling rather than oxidative damage, where the H₂O₂ functions as a secondary messenger that regulates the pathways for germination and growth^115–117^. This means that MF modulated the redox signaling cascades by which the ROS acted as signaling molecules, not as stress byproducts, and the Catalase activity reflected an adaptive detoxification. The progressive increase in amylase activity (1.55 → 2.09) under the various MF treatments indicated an induction of the starch hydrolysis, a critical process for energy supply during early germination. MF-mediated activation of hydrolytic enzymes has been repeatedly associated with improved seed vigor and metabolic flux toward glycolysis and respiration^23,118,119^. This means that MF enhances carbon mobilization, germination metabolism, energy availability, and stress adaptation. The changes in the protease activity, particularly the reduction at the DC and the enhancement at the 15 Hz, suggest a selective protein turnover during MF stress adaptation. The proteases play essential roles in the degradation of storage, damaged, and regulating signaling peptides during stress responses^120–122^. This means that as the stress-responsive proteolysis was activated, the MF will affect the protein quality control, and the developmental remodeling is facilitated. The strong fluctuation in the expression of the actin gene (170–924) under the MF treatments suggests a cytoskeletal remodeling, a known response to physical stimuli and ROS signaling. Actin dynamics are sensitive to electromagnetic fields; however, they regulate vesicle trafficking, cell elongation, and polarity during germination^123–125^. This means that MF primarily targets the cytoskeleton, where the cellular architecture is actively reorganized to cope with growth directionality. The observed fluctuation of *β*-actin at the transcript level (Fig. 6) suggests a change in actin-related processes that may include organelle movement and cellular reorganization during environmental stress^126–128^. The interaction between magnetic fields and cellular components may involve these actin-mediated structural pathways, complementing the biochemical and molecular changes observed in *sod* expression and antioxidant enzyme activity.

## Materials and methods

Certified aniseeds of *Pimpinella anisum* L. cv. “Baladi 1” (the local landrace commonly cultivated in Egypt) were used in this study. These seeds were obtained from the Medicinal and Aromatic Plants Research Department, Horticultural Research Institute, Agricultural Research Center, Giza, Egypt. These seeds were verified for purity and viability before exposure to magnetic treatments. Generally, this cultivar is commonly cultivated for its aromatic seeds and essential oil content and is known for its moderate germination rate and sensitivity to environmental conditions during early seedling establishment, making it a suitable model for evaluating seed priming and stress-related responses.

### Installation of the magnetic field apparatus

**Fig. 7:**
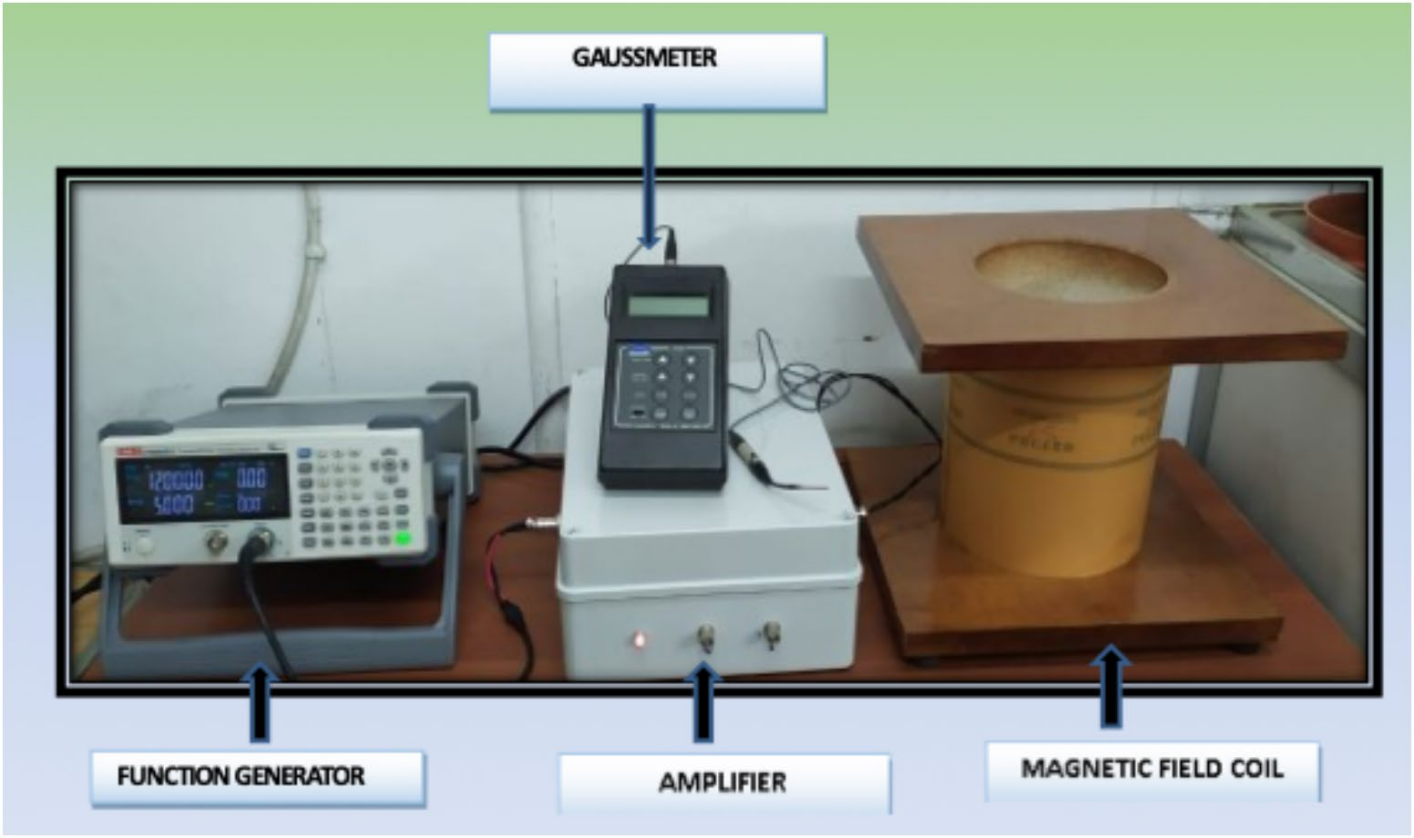
The MF exposure room and installment units.

The MF was generated using the four units: waveform function generator, power amplifier with a maximum 40 Gauss of MF strength, coil, and digital Gauss meter (Fig. 7). The details of the magnetic field apparatus were described by^129^. Briefly, the waveform function generator unit is the leading equipment used to provide electric currents that generate the MF (Uni-T, UTG9005c-II, Germany). The power amplifier unit is the output unit of the function generator that is connected to a power amplifier, which is based on a circuit of operational amplifiers, to amplify the signal to the required level of MF strength. The coil, which is made of 1000 turns of copper wires, with a resistance of about 3Ω, is the main unit responsible for the generation of the MF. The digital Gauss meter unit is used to monitor the strength of the MF produced by the coil in the sample exposure room (F.W. Bell, Model 4048, USA). Field homogeneity was assessed prior to experimentation using a calibrated Gaussmeter by mapping the magnetic flux density at multiple points across the sample exposure area. Measurements confirmed that the magnetic field intensity was maintained at 20 ± 0.5 Gauss within the central exposure zone, corresponding to a spatial variation of less than ± 2.5%. Simultaneously, samples were positioned within this central region to ensure uniform magnetic field exposure throughout the experimental period.

### Experimental design

A sterilized seeds were divided into 13 groups (100 seeds/group). Each group was exposed to MF strength 20 Gauss with different frequencies: DC (0 Hz), 5 Hz, 10 Hz, and 15 Hz. The MF was applied to each group at three exposure times (30, 60, and 90 min). The control group consisted of the same number of seeds that had not been treated with MF. The experiment was laid out in a complete randomized design with three replicates. Samples and data were collected after 1, 2, and 3 weeks.

### Germination assessment

A total of twenty-five sterilized magneto priming seeds were germinated in each sterilized Petri dish (9 cm diameter) covered at the bottom with two Whatman filter papers (No. 11), with a total of 52 dishes. Each Petri plate was watered using 5 ml of dd H_2_O, then sealed with Parafilm to retain moisture and incubated at 25°C and relative humidity (52.5%) for 8/16 h of light/dark rounds according to the International Seed Testing Association^130^. Four germination parameters were assessed. The germination percentage (G%) was calculated daily using the following equation: G% = (Number of germinated seeds/Total number of planted seeds)*100, following^131,132^. The vigor index (VI) was calculated according to^133^ to determine the strength and vitality of the seedlings following the equation: VI = [average lengths of seedlings’ root + shoot (cm)]*G%. Seedling root and shoot length (cm) were measured at weekly intervals (1st, 2nd, and 3rd week) to track growth dynamics over time. Based on the experimental results for germination assessment, the 90-min exposure duration was selected as the optimal representative group for all subsequent enzymatic and molecular studies. This exposure duration consistently yielded the highest germination percentage and long-term enhancement to seedling vigor across all frequencies compared to the control.

### Physiological assessment

Different enzyme activities, including α-amylase (AMY), protease (PRO), and catalase (CAT), were assayed in the 3-week-old seedlings. The amylase enzyme activity was measured using the starch method described by^134^. Furthermore, the protease enzyme activity was determined using the casein method^135^. Whereas the catalase enzyme activity was measured following the method of^136^ at 240 nm, reflecting the decomposition of H_2_O_2_. The enzyme activities were expressed as U/g of fresh weight.

### Expression of the antioxidant superoxide dismutase 1 (*sod*) and the cytoskeletal actin genes at 90 min exposure time

#### RNA and cDNA synthesis

Plant samples exposed to all MF treatments for 90 min were selected for RNA, cDNA, and further molecular assessment. The *sod* gene was amplified using the (*slsod1*) primers [Forward-(5′-AACCTGGACTTCATGGCTTC-3′) and Reverse-(5′-CCAGCAGGATTGTAATGTGG-3′)] presented by^137^. The RNA was extracted using the Qiagen RNeasy Plant Mini Kit (Qiagen, USA). RNA was purified to remove the genomic DNA by mixing one microgram of RNA in 1X reaction buffer [100 mM Tris–HCl (pH 7.6), 25 mM MgCl_2_, 5 mM CaCl_2_. 1 ml of 1 M Tris–HCl (pH 7.5)], 5 mM of MgCl_2_, 1 μl of DNase I enzyme, and 10 μl of DEPC-treated H_2_O, and incubated at 37 °C for 30 min, then the reaction was stopped by heating at 75°C for 15 min and addition of 5 mM EDTA (pH 8). The first cDNA was synthesized using one microgram of RNA that was preheated at 90 °C for two minutes, then 1X of 5X M-MLV reaction buffer, 2 mM of each dNTP (ATP, dCTP, dGTP, and dTTP), 1 μl of M-MLV-RT enzyme (200 U/ µL), and endonuclease-free H_2_O were mixed to a final volume of 25 µL. The mixture was incubated for 1 h at 37 °C. The reaction was stopped by heating the mixture at 70 °C for 15 min.

#### Quantification and RT-PCR-based amplification of the *sod* and actin genes

The two-step semi-quantitative RT-PCR method was used to determine gene expression in the previously built cDNA fragments. The PCR reaction mixture (25 μL) contained cDNA (2 μL), dNTPs (0.2 mM each), forward and reverse primers (0.4 mM each), MgCl_2_ (1.5–2 mM), 1X PCR buffer, and Taq DNA polymerase (1U/reaction). The PCR reaction was performed with an initial denaturation at 94 °C for 4 min, followed by 35 cycles of 94 °C for 30 s, then 50–55 °C for 30 s, and 72 °C for 30 s, with a final extension at 72 °C for 5 min. The PCR products were separated using agarose gel electrophoresis (2% in TAE buffer). The primers of *β*-actin were as follows: forward (5′-ATCCGGTCAGCAATACCAGG-3′) and reverse (5′-TTGCCCTGAGGTTCTGTTCC-3′) primers according to^138^. Quantitative estimates of amplified fragments’ intensities and numerical values of gene fold change were obtained using the Image Lab 6.1 software (Bio-Rad) and Antiabong et al.^139^ and Hazman et al.^140^. The T-test (two-tailed at α = 0.05) was used to signify the RT-PCR expression levels using the differential raw values of the band intensities in comparison with the values of the control samples. The t-test formula: t = [(treatment_mean)  − (control_mean)]/[(SD/√n)]. The number of replicates was n = 3, while the SD and mean values were calculated using the Excel software.

### Statistical analysis

All collected data were averaged using the arithmetic mean tool of Excel software. The means separation was applied only among significantly different treatments using the least significant differences (*LSD* at *p* > 0.05) revealed by the ANOVA output (Supplementary file). ANOVA, *Pearson’s* coefficient of correlation (*r*), and regression analysis were all conducted using the GenStat 17^th^ edition software (VSN International, 2017;^141^).

## Conclusion

MF treatments (DC, 5, 10, and 15 Hz) at exposure times of 30, 60, and 90 min significantly enhanced germination traits, physiological vigor, and molecular responses in aniseed (*Pimpinella anisum* L.), one of the most important aromatic plants. Results showed optimal regimes, with 5–10 Hz maximizing germination parameters and vigor. Enzyme activities increased in a frequency-specific manner, with catalase reaching high values at 10 Hz and α-amylase and protease exceeding untreated controls at 15 Hz. Molecular validation confirmed SOD down-regulation as MF strength increased. This suggests that MF acted as an abiotic stressor targeting ROS and elicited a non-linear, frequency-specific response. Thus, the antagonistic regulation of SOD and catalase suggests redox fine-tuning and signals adaptive ROS management rather than oxidative damage, with H₂O₂ (ROS) functioning as a secondary signaling regulator in pathways responsible for germination and growth, and catalase adapting as a detoxifying agent. Integrated findings revealed that MF-driven coordination of antioxidant defenses (catalase/SOD), hydrolytic metabolism (α-amylase/protease), and stress signaling aligns with plant developmental pathways that involve redox balance and cytoskeletal dynamics. Cytoskeletal remodeling was supported by up-regulation of the actin gene as MF strength increased, which targeted and reorganized cellular architecture to cope with growth directionality (vesicle trafficking, cell elongation, and polarity). The study identified 5–10 Hz as a “biologically active” window that offers superior, sustainable treatment compared with chemical methods. Expanding on this, the study paved the way for future research and implications supporting MF frequency for crop resilience, including: (1) testing optimal regimes (e.g., 5–10 Hz/60 min) across other plant species under open-field conditions; (2) investigating proposed physiological and functional mechanisms via multi-omics approaches (e.g., ROS signaling, Ca^2^⁺ fluxes); and (3) evaluating synergies/interactions when other stresses (drought + heat) are integrated for commercial seed technologies.

## Supplementary Information

Supplementary Information 1.

Supplementary Information 2.

## Data Availability

All relevant data are within the paper.
