# Supplementary figures and images for: Insights into germination, physiological, and molecular changes in aniseeds induced by magnetic fields
Source: Sci Rep. 2026 Jun 23;16:19542. doi: 10.1038/s41598-026-54832-2 (PMC13291271; doi:10.1038/s41598-026-54832-2)

**Supplementary Data**

**Here below are the original blots images**

**Actin
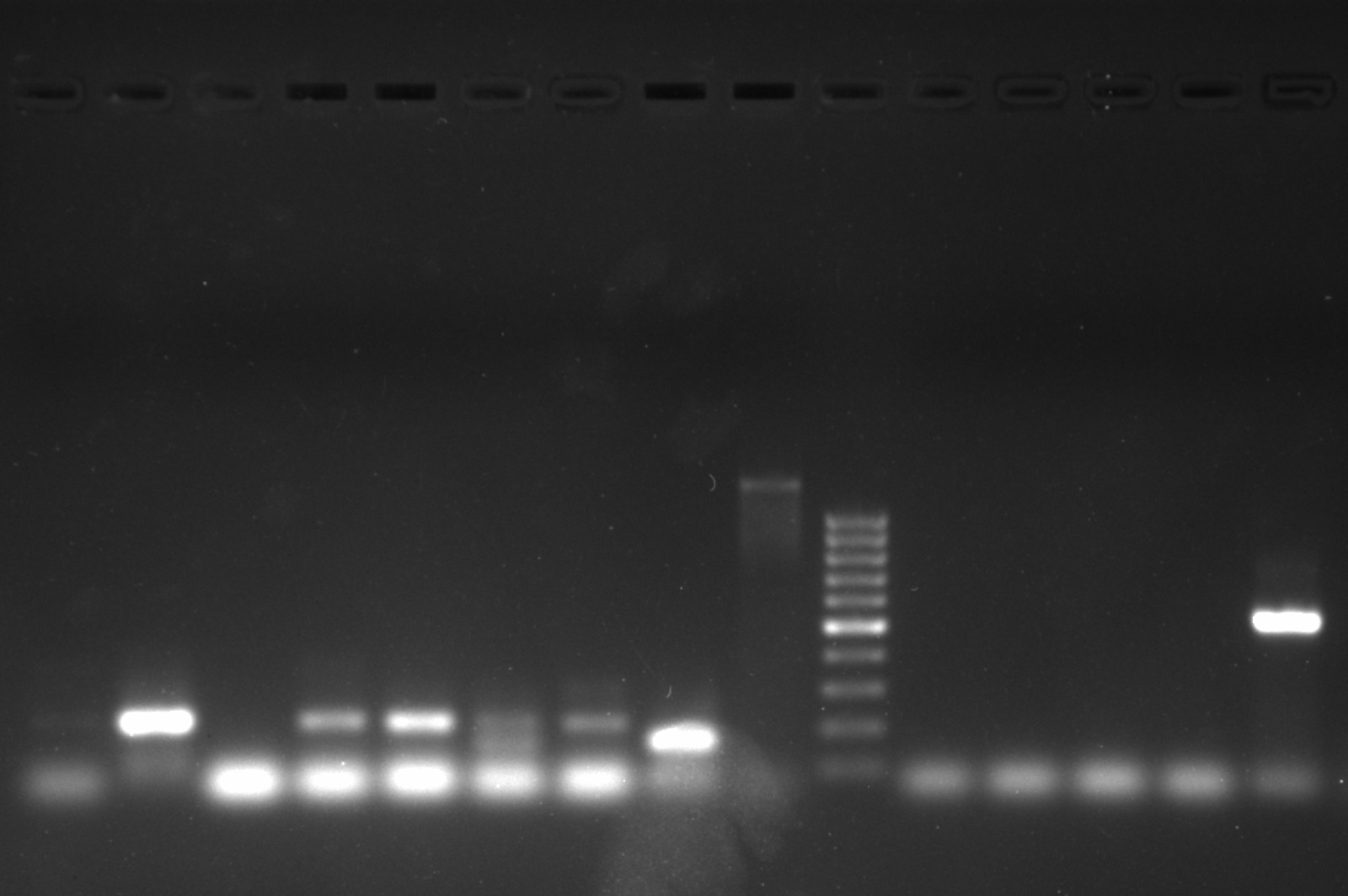
**

**5**

**DC**

**C**

**+ve**

**10**

**15**

**M**

**-ve**

**SOD**

**
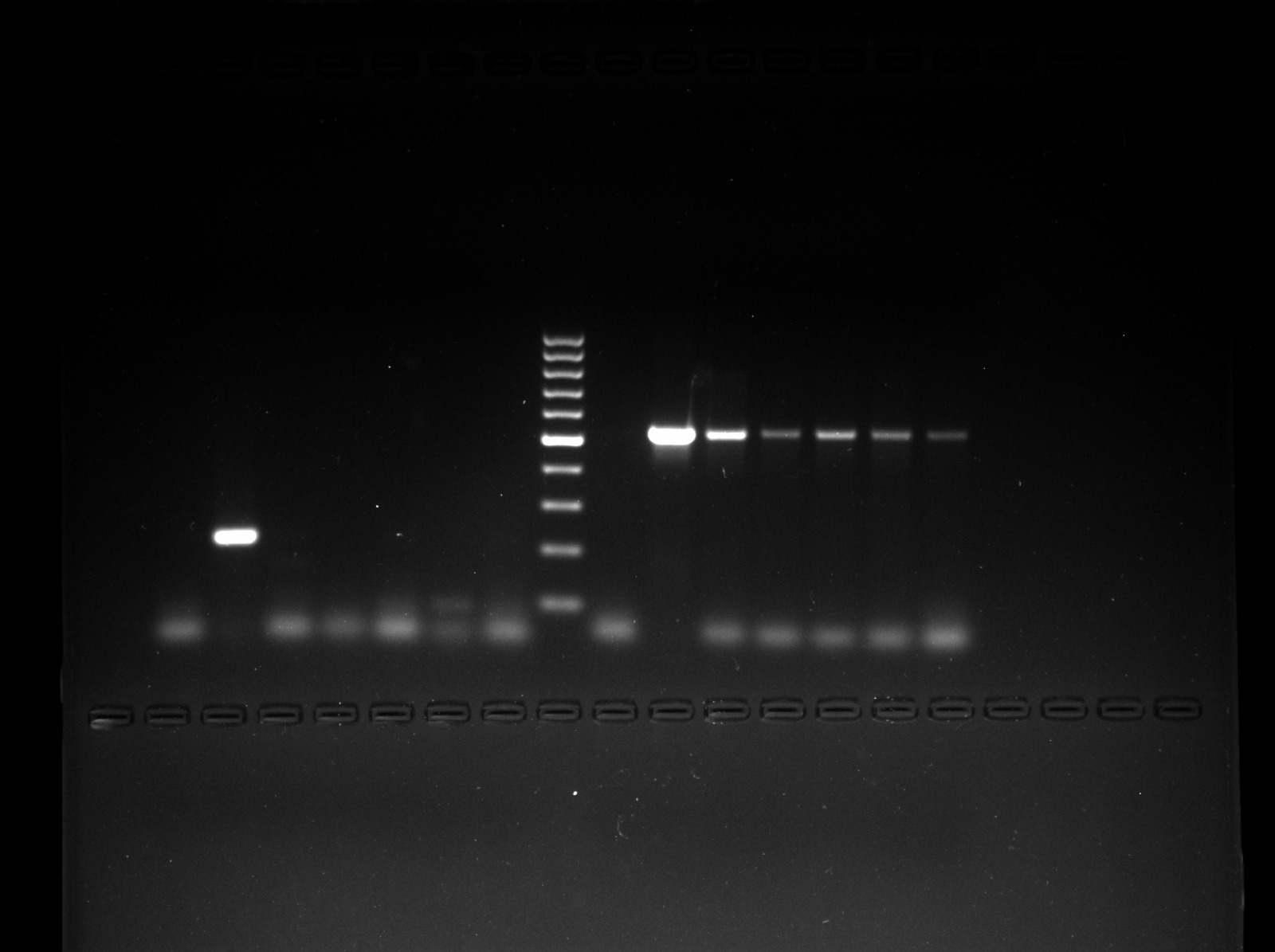
**

**10**

**15**

**5**

**DC**

**C**

**+ve**

**-ve**

**M**

Supplement: Supplementary file 2 — Supplementary Information 2. [file 41598_2026_54832_MOESM2_ESM.docx]
